# Hepatic Gene Expression and Metabolite Profiles of Androstenone and Skatole Relative to Plasma Estrone Sulfate Levels in Boars

**DOI:** 10.3390/biom14070850

**Published:** 2024-07-15

**Authors:** Christine Bone, E. James Squires

**Affiliations:** Department of Animal Biosciences, University of Guelph, Guelph, ON N1G2W1, Canada; cbone@uoguelph.ca

**Keywords:** boar taint, metabolism, androstenone, skatole, estrone sulfate, steroidogenic capacity

## Abstract

Testicular steroids can alter the activity and expression of enzymes within the liver and may influence the metabolism of skatole and androstenone, which are responsible for boar taint. Plasma levels of estrone sulfate (E_1_S) are indicative of the steroidogenic capacity of the boar and are variable between animals of similar live weights at slaughter. This study aimed to characterize the relationship between steroidogenic capacity and the metabolism of boar taint compounds by relating plasma E_1_S levels at slaughter weight to the expression levels of genes regulating the metabolism of androstenone and skatole, along with their respective metabolite profiles. RT-qPCR was used to evaluate gene expression in the liver. Hepatocytes were also isolated and treated with androstenone or skatole, with metabolite levels in the incubation media quantified by high-performance liquid chromatography. Plasma E_1_S levels ranged from 2.2–108.5 ng/mL and were positively correlated with overall skatole metabolism (*p* = 0.038), the production of metabolites 3-methyloxindole (*p* = 0.026) and 3-hydroxy-3-methyloxindole (*p* = 0.036), and expression levels of key genes involved in skatole metabolism, specifically *CYP2C33* (*p* = 0.0042), *CYP2C49* (*p* = 0.022), and *CYB5R1* (*p* = 0.017). There was no association between androstenone metabolism and plasma E_1_S concentrations; however, there was evidence of possible co-regulation amongst genes involved in the metabolism of androstenone, skatole, and estrogens. These findings indicate that steroidogenic capacity is related to the rate of skatole, but not androstenone metabolism, in slaughter-weight boars.

## 1. Introduction

Boar taint describes a meat quality issue characterized by an off-odor or off-flavor that develops in heated pork products from some entire male pigs. This results from the accumulation of androstenone (5α-androst-16-en-3-one), a testicular 16-androstene sex pheromone, and skatole (3-methylindole), a gut-derived metabolite of tryptophan, in the adipose tissue. The metabolism of androstenone and skatole is a two-phase process, primarily occurring in the liver, that involves a series of oxidation, reduction, or hydrolysis reactions (Phase I) followed by subsequent conjugation to a sulfonate group or glucuronic acid (Phase II).

Phase I metabolism of androstenone is regulated by two members of the aldo-keto reductase 1C (AKR1C) subfamily, AKR1C1 and AKR1C4, which are 3β- and 3α-hydroxysteroid dehydrogenase (HSD) enzymes that reduce androstenone to 3β- and 3α-androstenol, respectively [1,2]. The Phase I metabolites of skatole include 3-hydroxy-3-methylindolenine, 3-methyloxindole (3MOI), 2-aminoacetophenone (2AAP), 3-hydroxy-3-methyloxindole (HMOI), indole-3-carbinol (I3C), 5-hydroxy-3-methylindole (5-OH-3MI), and 6-hydroxy-3-methylindole (6-OH-3MI) [3,4,5]. These metabolites are produced from oxidation reactions mediated by several heme-containing monooxygenase enzymes belonging to the cytochrome P450 (CYP450) superfamily; mainly CYP2E1 and CYP2A19, and, to a lesser extent, CYP1A1, CYP2C49, CYP2C33v4, and CYP3A [5,6,7,8]. During Phase II metabolism, androstenone and its metabolites are sulfoconjugated by the sulfotransferase enzyme SULT2A1 [9], while SULT1A1 facilitates the sulfoconjugation of the hydroxy skatole metabolites 5-OH-3MI and 6-OH-3MI [10]. These metabolites can alternatively be glucuronidated by UDP-glucuronosyltransferases (UGTs).

Testicular steroids have been reported to influence the expression [11,12,13,14] and activity [15,16,17,18] of enzymes responsible for the metabolism of androstenone and skatole. This suggests that the hepatic metabolism rate of boar taint compounds may differ between animals with high and low capacities for steroidogenesis. Estrone sulfate (E_1_S) is a prominent steroid within the peripheral circulation of the boar [19] and there is notable variability in plasma E_1_S concentrations among boars of similar live weights at slaughter [20]. Plasma E_1_S levels are considered a proxy for overall steroid hormone levels and thus reflect the steroidogenic capacity of the boar [21,22]. This is further evidenced by the positive relationship identified between plasma levels of androstenone, E_1_S, and fat androstenone concentrations at slaughter [20]. 

Estrogen receptor α has been shown to regulate the hepatic expression of several CYP450s in humans, both in response to and in the absence of estrogens [23,24], and both 17β-estradiol and estrone were previously shown to inhibit CYP2E1 activity in entire male, but not female, pigs [16,17]. However, the implications of these findings for the metabolism of boar taint compounds are unclear, given that the effect of these hormone-related differences in enzyme expression and activity on the metabolism of androstenone and skatole within the liver has not been evaluated. Hence, this study related plasma E_1_S levels at slaughter to the expression levels of genes regulating the metabolism of androstenone and skatole and their respective metabolite profiles in isolated hepatocytes. Our objective was to elucidate the interplay between steroidogenic capacity, as indicated by plasma E_1_S levels, and the metabolism of androstenone and skatole in slaughter-weight boars.

## 2. Materials and Methods

### 2.1. Animals and Sample Collection

Terminal cross [Duroc × (Landrace × Yorkshire)] boars (*n* = 8) were housed in groups of 4 beginning around 10 weeks of age and were provided ad libitum access to water and standard starter, grower, and finisher rations (Flordale Feed Mill Limited, Floradale, ON, Canada). At 187.3 ± 6.9 days of age and approximately 140 kg live weight, the boars were electrically stunned and exsanguinated, with samples of liver and blood collected from each animal. All animals were used in accordance with the guidelines of the Canadian Council of Animal Care and the University of Guelph Animal Care Policy. Liver lobes were used immediately for the isolation of hepatocytes and additional liver samples were stored at −80 °C for later analysis of gene expression. Plasma E_1_S levels were quantified as an indicator of steroidogenic capacity using an E_1_S-specific radioimmunoassay [25].

### 2.2. Hepatocyte Isolation and Treatments

Hepatocytes were isolated as previously described by Gray and Squires [26] with modifications. Briefly, the liver lobe was canulated and perfused with a blanching solution (10% Hank’s balanced salt solution (10×, no Ca^2+^, Mg^2+^, HCO_3_^−^, or phenol red), 10 mM HEPES, 1 mM EGTA, pH 7.4) for 10 min at a flow rate of 25 mL/min, followed by a rinsing buffer (10% Hank’s balanced salt solution, 10 mM HEPES, pH 7.4) for 10 min. The lobe was then perfused with a digestion buffer (10 mM HEPES and 0.71 mg/mL collagenase type I in William’s media E, pH 7.4) for 30 min at a flow rate of 20 mL/min. Following digestion, the liver was dissected and the liberated hepatocytes were collected in attachment media (10 mM HEPES, 12.1 nM insulin from bovine pancreas, 10% fetal bovine serum, and 1% penicillin/streptomycin in William’s media E, pH 7.4) and filtered through a 255 µm nylon mesh into 50 mL conical tubes. The cells were then centrifuged at 100× *g* for 3 min and rinsed twice with fresh attachment media to remove the collagenase remaining from the digestion buffer. After rinsing, the cells were counted using a hemocytometer, and a 0.04% trypan blue exclusion test was used to determine cell viability, which was typically 90% or greater. Cells were suspended in attachment media and plated at a seeding density of 0.5 million cells/well in 24-well standard surface-treated polystyrene tissue culture plates (Fisher Scientific, Toronto, ON, Canada).

The attachment media was removed from the cells 4 h after plating and replaced with 0.5 mL serum-free media (10 mM HEPES, 12.1 nM insulin from bovine pancreas, 10 mM pyruvate, 0.35 mM L-proline, and 1% penicillin/streptomycin in William’s media E, pH 7.4) containing 0.05% (*v*/*v*) dimethyl sulfoxide (DMSO) or 0.1% ethanol vehicle for 20 h. The media was then replaced with 0.5 mL serum-free media containing [^3^H]-androstenone (20 µM, 13.5 µCi/µmol, 0.1% ethanol) or skatole (200 µM, 0.05% (*v*/*v*) DMSO). The treatments were performed in triplicate and incubations with androstenone were terminated at 0, 1, 2, 3, 4, 6, and 8 h post-treatment while incubations with skatole were terminated at 0, 2, 4, 8, and 12 h post-treatment. 

### 2.3. Assessment of Metabolite Production by HPLC

The media from each incubation was diluted 1:1 (*v*/*v*) with 100% acetonitrile, centrifuged at 8000× *g* for 10 min, then filtered with a 0.2 µm nylon syringe filter (Fisher Scientific, Toronto, ON, Canada). Metabolite production was quantified by reverse-phase C18 HPLC using a Luna 5 µm C18 HPLC column (250 × 4.60 mm) (Phenomenex, Torrance, CA, USA).

The metabolism of androstenone was assessed using a 40-min HPLC profile previously described by Laderoute et al. [9]. The elution of [^3^H] 3α/β- androstenol and androstenone was monitored using an online isotope detector and occurred at a retention time of approximately 33 and 34 min, respectively. A peak with a retention time of 3 min was identified as 16-androstene glucuronides as follows: the peak was collected and incubated overnight at 37 °C in 1 mL sodium acetate (0.5 M, pH 5.0) containing 2500 units/mL of β-glucuronidase (type B-1, from bovine liver). Following β-glucuronidase treatment, the fraction was analyzed by HPLC using the aforementioned 40-min profile, and two peaks were detected at 33 and 34 min, which matched the retention time of 3α/β-androstenol and androstenone, respectively. 

Skatole metabolism was quantified using a 35-min HPLC profile modified after Wiercinska et al. [5] using a solvent system consisting of buffer A: 90% 5 mM potassium dihydrogen phosphate (pH 3.9) and 10% acetonitrile (*v*/*v*); buffer B: 100% acetonitrile. The flow rate was 1 mL/min and the gradient profile was as follows: 0–10 min, 90% buffer A and 10% buffer B; 10–25 min, 70% buffer A and 30% buffer B; 25–25.1 min, 30% buffer A and 70% buffer B; 25.1–30.1 min, 0% buffer A and 100% buffer B; 30.1–35 min, 90% buffer A and 10% buffer B. The elution of 6-OH-3MI glucuronide and I3C occurred at approximately 6 and 10 min, respectively, and was monitored by fluorescence detection with an excitation wavelength of 285 nm and emission wavelength of 350 nm. UV absorbance at 250 nm was used to monitor the elution of HMOI, 3MOI, and skatole, which occurred at approximately 7, 13, and 24 min, respectively.

### 2.4. RNA Extraction and Evaluation of Gene Expression by Real-Time qPCR

Frozen liver samples collected at slaughter (30 mg) were homogenized in 600 µL lysis buffer and RNA was extracted using the RNeasy Mini Kit (Qiagen, Valencia, CA, USA) according to the manufacturer’s instructions. The RNA concentration and integrity were assessed using a NanoDrop 8000 spectrophotometer (Thermo Scientific, Waltham, MA, USA) and an Agilent 2000 Bioanalyzer (Agilent Technologies, Santa Clara, CA, USA), respectively. The RNA was reverse transcribed, and the resulting cDNA was amplified by RT-qPCR as previously described by Bone and Squires [22] to evaluate the expression levels of key gene transcripts associated with androstenone, skatole, and estrogen metabolism (Table 1) using the primers listed in Table 2. RT-qPCR reactions were run in triplicate and gene expression was calculated using the 2^−∆∆Ct^ method [27] using β-actin as a housekeeping gene. For each gene of interest, the expression relative to β-actin was averaged across all boars and used as the calibrator to calculate relative fold change.

### 2.5. Statistical Analysis

Statistical analysis was performed in SAS 9.4 (SAS Institute, Cary, NC, USA). Pearson correlation coefficients were calculated to evaluate the relationships between hepatic gene expression levels, metabolite profiles, and plasma levels of E_1_S. Correlation coefficients were calculated with the following model and considered statistically significant at *p* < 0.05:

$$\rho =\frac{\sigma_{xy}}{\sqrt{\sigma_{x}^{2}\sigma_{y}^{2}}}$$

where $\sigma_{x}^{2}$ is the variance of the *x* variable, $\sigma_{y}^{2}$ is the variance of the *y* variable, and *σ_xy_* is the covariance between *x* and *y*.

## 3. Results

### 3.1. Time Course Analysis of Androstenone and Skatole Metabolism by Hepatocytes

**Figure 1:**
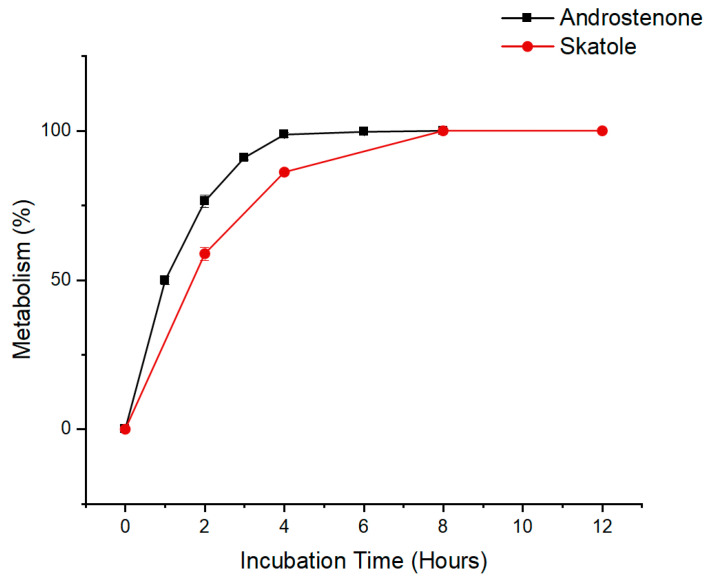
Time course data for the metabolism of androstenone and skatole in isolated porcine hepatocytes. Hepatocytes were incubated with androstenone for 0, 1, 2, 3, 4, 6, and 8 h or skatole for 0, 2, 4, 8, and 12 h. Media from each time point was analyzed by HPLC to determine the metabolism of androstenone and skatole over time. Data are presented as the mean ± standard error from 3 technical replicates.

Time course data for the overall metabolism of androstenone and skatole by primary porcine hepatocytes is shown in Figure 1 and represents the disappearance of each substrate over time. The overall metabolism of androstenone was rapid and increased over time from 49.9 ± 2.0% after 1 h to 90.9 ± 1.5% after 3 h. Androstenone metabolism plateaued after 4 h with marginal increases in metabolism occurring thereafter. In contrast, overall skatole metabolism increased more gradually over time, from 58.8 ± 2.2% after 2 h to 86.2 ± 0.2% after 4 h, with a plateau in metabolism after 8 h. Based on these results, an incubation time of 3 h was determined optimal for quantifying the metabolism of both androstenone and skatole.

### 3.2. Assessment of Hepatic Gene Expression Levels and Metabolite Profiles of Androstenone and Skatole in Slaughter-Weight Boars

Hepatic expression levels of key genes responsible for the metabolism of boar taint compounds were quantified using RT-qPCR. Gene expression levels were then related to the overall metabolism of androstenone and skatole and the production of their corresponding metabolites in isolated hepatocytes, which were expressed as a percentage of the total compounds detected. The overall metabolism of substrate varied between individual boars ranging from 64.30–95.40% for androstenone (Table 3A) and 16.57–76.84% for skatole (Table 3B).

**Figure 2:**
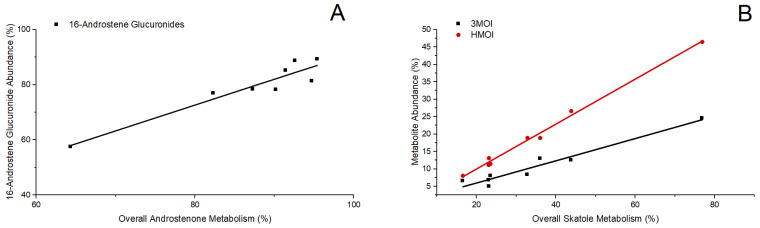
Relationship between (**A**) overall androstenone metabolism and production of 16-androstenes (r = 0.95, *p* = 0.0004); (**B**) overall skatole metabolism and the production of HMOI (r = 0.99, *p* < 0.0001) and 3MOI (r = 0.97, *p* < 0.0001).

Androstenone metabolism in isolated hepatocytes resulted in the production of Phase I metabolites 3α/β-androstenol and Phase II 16-androstene glucuronide conjugates. The overall metabolism of androstenone quantified was positively correlated with the production of 16-androstene glucuronides (r = 0.95, *p* = 0.0004, Figure 2A), which were the most abundant metabolites of androstenone metabolism (Table 3A). In contrast, the synthesis of 3α/β-androstenol was neither correlated with the overall metabolism of androstenone nor the production of 16-androstene glucuronides. These results suggest that 3α/β-androstenol may primarily serve as metabolic intermediates in hepatic androstenone metabolism.

Skatole metabolism yielded the Phase I metabolites HMOI, 3MOI, and I3C and the Phase II metabolite 6-OH-3MI glucuronide. HMOI was the most abundant metabolite of skatole produced followed by 3MOI, 6-OH-3MI glucuronide, and I3C (Table 3B). Overall, skatole metabolism in isolated hepatocytes was positively correlated (Figure 2B) with the production of HMOI (r = 0.99, *p* < 0.0001) and 3MOI (r = 0.97, *p* < 0.0001), but was not significantly associated with the synthesis of either I3C or 6-OH-3MI glucuronide. However, I3C synthesis was positively correlated with the overall metabolism of androstenone (r = 0.72, *p* = 0.042), suggesting potential co-regulation among genes mediating the production of I3C and hepatic androstenone metabolism.

The mean relative fold change quantified for each gene of interest is presented in Table 4, along with the range in expression levels seen across individual boars. Expression levels of key genes involved in regulating the metabolism of boar taint compounds were not correlated with the overall metabolism of androstenone or skatole, nor with the production of their respective metabolites. These results likely reflect the combined effect of different AKR1Cs and CYP450s on the metabolism of androstenone and skatole, respectively, which was not evaluated in the present study.

### 3.3. Relationship between Plasma E_1_S Levels and the Metabolism of Boar Taint Compounds

Plasma E_1_S levels quantified by RIA were used as a proxy for overall steroid hormone levels and related to the expression levels of genes regulating the hepatic metabolism of estrogens and boar taint compounds, as well as the metabolite profiles of androstenone and skatole in slaughter-weight boars. This enabled the relationship between steroidogenic capacity and boar taint metabolism to be assessed and the potential interplay between the metabolism of estrogens, androstenone, and skatole to be characterized.

**Figure 3:**
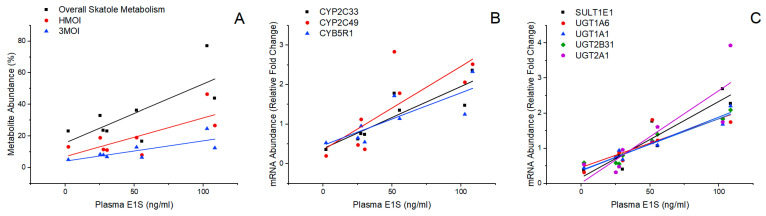
Relationship between plasma E_1_S levels and (**A**) overall skatole metabolism (r = 0.73, *p* = 0.038), HMOI production (r = 0.74, *p* = 0.036), and 3MOI production (r = 0.77, *p* = 0.026); (**B**) expression levels of *CYP2C33* (r = 0.88, *p* = 0.0042), *CYP2C49* (r = 0.78, *p* = 0.022), and *CYB5R1* (r = 0.80, *p* = 0.017); (**C**) expression levels of *SULT1E1* (r = 0.94, *p* = 0.0006), *UGT1A6* (r = 0.88, *p* = 0.0035), *UGT1A1* (r = 0.97, *p* < 0.0001), *UGT2B31* (r = 0.97, *p* < 0.0001), and *UGT2A1* (r = 0.85, *p* = 0.0069).

Plasma levels of E_1_S varied considerably between individual animals ranging from 2.2–108.5 ng/mL with a mean of 50.5 ± 13.4 ng/mL and were correlated with metabolite (Figure 3A) and gene expression levels (Figure 3B) related to skatole metabolism. Notably, positive correlations were observed with overall skatole metabolism (r = 0.73, *p* = 0.038), HMOI production (r = 0.74, *p* = 0.036), 3MOI production (r = 0.77, *p* = 0.026), *CYP2C49* expression (r = 0.78, *p* = 0.022), *CYP2C33* expression (r = 0.88, *p* = 0.0042), and *CYB5R1* expression (r = 0.80, *p* = 0.017). Associations between plasma E_1_S levels and expression levels of *CYP2E1*, *CYP2A19*, and *SULT1A1* were not statistically significant. Additionally, plasma E_1_S levels were not correlated with the overall metabolism of androstenone, the production of androstenone metabolites, or the expression of genes regulating androstenone metabolism. These results demonstrate that steroidogenic capacity, as indicated by plasma E_1_S levels, is related to the metabolism of skatole, but not androstenone, in slaughter-weight boars.

**Figure 4:**
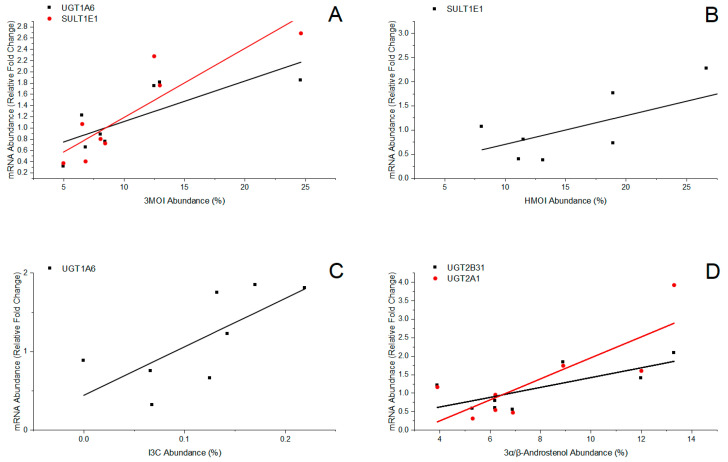
Relationship between (**A**) 3MOI production and expression levels of *UGT1A6* (r = 0.77, *p* = 0.024) and *SULT1E1* (r = 0.89, *p* = 0.0031); (**B**) HMOI production and expression levels of *SULT1E1* (r = 0.84, *p* = 0.0084); (**C**) I3C production and expression levels of *UGT1A6* (r = 0.72, *p* = 0.045); (**D**) 3α/β-androstenol production and expression levels of *UGT2B31* (r = 0.73, *p* = 0.039) and *UGT2A1* (r = 0.80, *p* = 0.016).

Plasma E_1_S levels were strongly correlated with the expression of several genes involved in Phase II estrogen metabolism (Figure 3C), including *SULT1E1* (r = 0.94, *p* = 0.0006), *UGT1A6* (r = 0.88, *p* = 0.0035), *UGT1A1* (r = 0.97, *p* < 0.0001), *UGT2B31* (r = 0.97, *p* < 0.0001), and *UGT2A1* (r = 0.85, *p* = 0.0069). Significant associations were also identified between 3MOI production (Figure 4A) and expression levels of *UGT1A6* (r = 0.77, *p* = 0.024) and *SULT1E1* (r = 0.89, *p* = 0.0031); HMOI production (Figure 4B) and expression levels of *SULT1E1* (r = 0.84, *p* = 0.0084); I3C production (Figure 4C) and expression levels of *UGT1A6* (r = 0.72, *p* = 0.045); and 3α/β-androstenol production (Figure 4D) and expression levels of *UGT2B31* (r = 0.73, *p* = 0.039) and *UGT2A1* (r = 0.80, *p* = 0.016). These findings may reflect co-regulation among genes involved in Phase II estrogen metabolism and those regulating the synthesis of 3MOI, HMOI, I3C, and 3α/β-androstenol.

## 4. Discussion

Boar taint is a multifactorial issue that is dependent on the difference between the rate of synthesis and metabolism of androstenone and skatole; this is influenced by several physiological, genetic, nutritional, and environmental factors, which determines the extent of their accumulation in the fat [35,36]. Plasma E_1_S levels reflect the steroidogenic capacity of the animal and are of special interest as they are highly related to the rate of androstenone synthesis and fat androstenone levels at slaughter weight [20]. Steroidogenic capacity is also thought to be an important physiological factor influencing the hepatic metabolism of boar taint compounds. Estrogens including estrone, 17β-estradiol, and 17α-estradiol were previously shown to inhibit CYP2E1 activity in pig liver microsomes from entire males in a sex- and breed-dependent manner [16,17,18]. Breed- and sex-dependent differences in the expression profiles of genes regulating the metabolism of boar taint compounds have also been described [11,12]. Therefore, this study related the hepatic gene expression and metabolite profiles of androstenone and skatole to plasma E_1_S levels in order to assess the relationship between steroidogenic capacity and the metabolism of boar taint compounds. 

Elevated levels of 6-OH-3MI sulfate and reduced levels of HMOI were previously identified in the plasma of boars exhibiting efficient skatole clearance [3]. Additionally, positive correlations have been observed between the hepatic activity of SULT1A1 in pre-pubescent boars and skatole concentrations in the plasma and fat [37]. Consequently, levels of 6-OH-3MI sulfate and HMOI were suggested as possible biomarkers for boars with low- and high-fat skatole levels, respectively. In the present study, HMOI and 3MOI emerged as the predominant metabolites of skatole metabolism, with isolated hepatocytes producing 6-OH-3MI glucuronide rather than 6-OH-3MI sulfate as a Phase II skatole metabolite. Moreover, overall skatole metabolism was positively correlated with the production of both HMOI and 3MOI, suggesting that these metabolites should be considered as potential biomarkers for the rate of skatole metabolism in slaughter-weight boars.

We also identified positive correlations between plasma levels of E_1_S and the expression levels of key genes (*CYP2C49*, *CYP2C33*, and *CYB5R1*) and metabolites (HMOI, 3MOI) associated with skatole metabolism. In contrast, expression levels of *CYP2E1*, *CYP2A19*, and *SULT1A1* tended to be lower in animals with elevated plasma E_1_S levels, but these relationships were not statistically significant. These findings suggest that the rate of skatole metabolism may differ between animals with high and low capacities for steroid hormone production, as indicated by their plasma E_1_S levels. This conclusion is further supported by the decreased hepatic expression levels of *CYP2E1*, *CYP2A19*, *SULT1A1*, and *SULT2A1* and increased expression levels of *CYP2C33*, *CYP2C49*, *UGT2B31*, *UGT1A1*, and *UGT1A6* that have been observed in breeds of boars with elevated serum concentrations of androgens compared to those with lower serum androgen levels [11,12,38]. Similarly, Moe et al. [39] reported that the hepatic expression of *CYP2C33* and *CYP2C49* was significantly increased in boars with high androstenone levels relative to low-androstenone boars. The relationship identified between plasma E_1_S levels and skatole metabolism in the present study may have important implications for the identification and treatment of boars with differing rates of skatole metabolism. Recent reports have highlighted differences in the regulation and prediction of boar taint development between early- and late-maturing boars, distinguished by their differences in plasma E_1_S levels at slaughter weight [24,25]. Therefore, our findings may be particularly useful for assessing differences in skatole metabolism between early- and late-maturing boars; however, additional research with a larger sample size is required to validate these results before evaluating the potential of E_1_S levels as a biomarker for the rate of skatole metabolism in vivo and establishing thresholds to differentiate between boars with high and low rates of metabolism.

Hepatic gene expression levels and metabolite profiles associated with androstenone metabolism were not related to plasma levels of E_1_S in the present study. E_1_S was previously reported to increase AKR1C1 protein expression in primary porcine hepatocytes isolated from boars with an average carcass weight of 92 kgs; however, this same effect was not observed in hepatocytes isolated from the livers of lighter-weight boars with an average carcass weight of 70 kgs [14]. Therefore, factors such as age and weight may have a greater impact on hepatic androstenone metabolism than the animal’s steroidogenic capacity, although co-regulation among genes involved in the metabolism of androstenone, skatole, and estrogens may also be a contributing factor. Our results provided evidence of co-regulation among genes involved in the synthesis of I3C and androstenone metabolism, as well as genes associated with Phase II estrogen metabolism and those responsible for the production of HMOI, 3MOI, I3C, and 3α/β-androstenol. Consistent with our findings, 17β-estradiol was previously found to induce *CYP2C49* expression in isolated porcine hepatocytes, and transactivation of the transcription factor pregnane X receptor (PXR) increased both I3C synthesis and androstenone metabolism [26]. In addition to PXR, there are several transcription factors capable of regulating the expression of genes involved in the metabolism of both estrogens and boar taint compounds, including constitutive androstane receptor (CAR), farnesoid X receptor (FXR), aryl hydrocarbon receptor (AhR), and specificity protein 1 (Sp1) [40,41,42,43,44,45]. Cross-talk between these transcription factors and estrogen receptor alpha (ERα) has been described in humans and mice [23,24,29,46], further supporting the gene co-regulation proposed in this study. Several fermentable carbohydrates and natural products have been suggested as potential dietary treatments for boar taint due to the active compounds they contain or produce, which act as ligands for transcription factors regulating the metabolism of boar taint compounds [47]. However, co-regulatory networks among genes responsible for the metabolism of androstenone, skatole, and estrogens have not been fully established, nor have the impact of age or plasma E_1_S levels on hepatic expression levels of these different transcription factors been assessed. Therefore, future research should evaluate the interplay among the different transcription factors regulating hepatic androstenone, skatole, and estrogen metabolism, using a larger sample size of boars with varying steroidogenic capacities. This is crucial for developing effective dietary interventions that prevent boar taint by targeting the gene co-regulatory networks responsible for androstenone and skatole metabolism, with the objective of increasing their rate of hepatic metabolism.

## 5. Conclusions

This study identified HMOI and 3MOI as potential biomarkers of skatole metabolism and suggested that steroidogenic capacity, indicated by plasma E_1_S levels, is related to the rate of skatole metabolism in slaughter-weight boars. Positive associations were observed between plasma E_1_S levels and the metabolite (HMOI and 3MOI) and hepatic gene expression (*CYP2C49*, *CYP2C33*, and *CYB5R1*) levels related to skatole metabolism. The hepatic metabolism of androstenone was not related to plasma levels of E_1_S; however, androstenone metabolism was positively correlated with I3C synthesis, as were expression levels of genes regulating estrogen metabolism with the production of HMOI, 3MOI, I3C, and 3α/β-androstenol. This suggests that the expression of genes responsible for androstenone, skatole, and estrogen metabolism may be regulated by common transcription factors. Additional research with a larger sample size is needed to validate the relationship between plasma E_1_S levels and the rate of skatole metabolism in vivo, as well as to characterize the mechanisms of gene co-regulation in boars with differing steroidogenic capacities.

## Figures and Tables

**Table 1:** Abbreviations, names, functions, and NCBI reference sequences (Refseq ID) for gene transcripts of interest.

Gene Abbreviation	Gene Name	Function	Refseq ID
*CYP2A19*	Cytochrome P450 2A19	Phase I skatole metabolism [5,6,7,8]	NM_214417
*CYP2E1*	Cytochrome P450 2E1		NM_214421
*CYP2C33*	Cytochrome P450 2C33		NM_214414
*CYP2C49*	Cytochrome P450 2C49		NM_214420
*CYB5A*	Cytochrome B5A	Phase I skatole metabolism and steroidogenesis [5,28]	XM_005666296
*CYBR1*	Cytochrome B5 reductase, type 1		NM_001243918.1
*CYBR3*	Cytochrome B5 reductase, type 3		XM_003125982.4
*AKR1C1*	Aldo-keto reductase 1C1	Phase I androstenone metabolism [2]	NM_001044618
*SULT1A1*	Sulfotransferase 1A1	Phase II sulfoconjugation of skatole [10]	NM_213765
*SULT2A1*	Sulfotransferase 2A1	Phase II sulfoconjugation of androstenone [9]	NM_001037150
*SULT1E1*	Sulfotransferase 1E1	Phase II sulfoconjugation of estrogens [29]	NM_213992.1
*UGT1A6*	UDP-glucuronosyltransferase 1A6	Phase II glucuronidation of estrogens [30,31,32,33,34]	NM_001278750.1
*UGT1A1*	UDP-glucuronosyltransferase 1A1		KJ922612.1
*UGT2A1*	UDP-glucuronosyltransferase 2A1		XM_003356958.4
*UGT2B31*	UDP-glucuronosyltransferase 2B31		NM_001244124.1
*ACTB*	β-Actin	-	XM_003357928

**Table 2:** Primer sequences used in real-time qPCR analyses.

Primer	Forward Sequence	Reverse Sequence
CYP2A19	5′-TGAACACGGAGCAGATGTACAAC-3′	5′-CTCCTTCACCGCGTCGTATC-3′
CYP2E1	5′-TGCTCCACTACAAGAATGAGTTCTCT-3′	5′-GGGAGAACCGCCGAGTGT-3′
CYP2C33	5′-TTGGATAAAGATGGCAGCTTCAG-3′	5′-AATGGTGGTGAAGAACAGGAAGA-3′
CYP2C49	5′-TCCCCAACCCAGAGGTGTT-3′	5′-CCTTCTCCCACACAAATTCGTT-3′
CYB5A	5′-AGTCCGACAAAGCCGTGAA-3′	5′-CACCTCCAGCTTGTTCCCT-3′
CYBR1	5′-ATTTCCTGAGGGAGGGAAGA-3′	5′-GGCTGAATGCTGAACTTTCC-3′
CYBR3	5′-GTGATGACGACAAGGGCTTT-3′	5′-AAACTTTCCTTTGCCCTGGT-3′
AKR1C1	5′-GGAGGACTTTTTCCCAAAGG-3′	5′-TCCCTCGTTCTTGCACTTCT-3′
SULT1A1	5′-GAACAACGCCATGACCAACTAC-3′	5′-GGTTACAGCCTGCCATCTTC-3′
SULT2A1	5′-ACACGAGAAGCGCCGTAGAG-3′	5′-TGGACATGTTGTTTTCTTTCATGA-3′
SULT1E1	5′-GCATCAGATGAGCTTGTGGA-3′	5′-AGTCTCCTGCAATCCCCTTT-3′
UGT1A6	5′-TGCTTTGGGCAAAATACCTC-3′	5′-CTTTGGGTGACCAAGCAGAT-3′
UGT1A1	5′-ATAATTACCCGAGGCCCATC-3′	5′-CCCCAAAGAGAAAACCACAA-3′
UGT2B31	5′-TTTGAGACAATGGGGAAAGC-3′	5′-AGGTAGGGGTTTTGCAGGTT-3′
UGT2A1	5′-TGCACGTTACTGAAAATGCAAG-3′	5′-TTGTAAAAGCCAGAGCACATCA-3′
ACTB	5′-CGTGGACATCAGGAAGGAC-3′	5′-TCTGCTGGAAGGTGGACAG-3′

**Table 3:** (**A**). Androstenone metabolite profile in isolated hepatocytes. (**B**). Skatole metabolite profile in isolated hepatocytes.

**(A)**		
	**Metabolite Abundance (%)**	
**Metabolite**	**Mean ± Standard Error**	**Range**
16-Androstene Glucuronides	79.45 ± 3.56	57.50–89.30
Androstenols	7.84 ± 1.17	3.90–13.30
Overall Androstenone Metabolism	87.28 ± 3.60	64.30–95.40
Values shown represent the percentage of each metabolite quantified by high-performance liquid chromatography in the media from isolated hepatocytes following 3-h incubations with androstenone (*n* = 8).		
**(B)**		
	**Metabolite Abundance (%)**	
**Metabolite**	**Mean ± Standard Error**	**Range**
HMOI	19.35 ± 4.40	8.06–46.45
3MOI	10.61 ± 2.23	4.98–24.61
6-OH-3MI Glucuronide	4.40 ± 0.43	1.84–5.61
I3C	0.12 ± 0.02	0.00–0.22
Overall Skatole Metabolism	34.46 ± 6.80	16.57–76.84
Values shown represent the percentage of each metabolite quantified by high-performance liquid chromatography in the media from isolated hepatocytes following 3-h incubations with skatole (*n* = 8).		

**Table 4:** Expression levels in liver tissue for gene transcripts of interest.

	Gene Expression (Relative Fold Change)	
Gene	Mean ± Standard Error	Range
*CYP2A19*	18.60 ± 11.63	0.01–89.11
*CYP2C33*	1.18 ± 0.24	0.36–2.36
*CYP2E1*	4.59 ± 2.08	0.01–15.86
*CYP2C49*	1.42 ± 0.36	0.20–2.83
*AKR1C1*	1.18 ± 0.23	0.28–2.33
*CYB5A*	1.25 ± 0.36	0.36–3.16
*CYB5R1*	1.14 ± 0.22	0.53–2.32
*CYB5R3*	1.32 ± 0.37	0.51–2.75
*SULT1A1*	2.08 ± 0.87	0.30–6.58
*SULT2A1*	1.18 ± 0.21	0.25–1.98
*SULT1E1*	1.26 ± 0.31	0.40–2.69
*UGT1A6*	1.16 ± 0.21	0.32–1.85
*UGT1A1*	1.12 ± 0.20	0.44–2.20
*UGT2B31*	1.13 ± 0.21	0.56–2.09
*UGT2A1*	1.34 ± 0.41	0.31–3.92

## Data Availability

The original contributions presented in the study are included in the article, further inquiries can be directed to the corresponding author.
